# Transcriptomic Analysis of Dark-Induced Senescence in Bermudagrass (*Cynodon dactylon*)

**DOI:** 10.3390/plants8120614

**Published:** 2019-12-17

**Authors:** Jibiao Fan, Yanhong Lou, Haiyan Shi, Liang Chen, Liwen Cao

**Affiliations:** 1 College of Animal Science and Technology, Yangzhou University, Yangzhou 225009, China; 006298@yzu.edu.cn; 2 CAS Key Laboratory of Plant Germplasm Enhancement and Specialty Agriculture, Wuhan Botanical Garden, Chinese Academy of Sciences, Wuhan 430074, China; 3 Center of Economic Botany, Core Botanical Gardens, Chinese Academy of Sciences, Wuhan 430074, China; 4 National Engineering Laboratory for Efficient Utilization of Soil and Fertilizer Resources, College of Resources and Environment, Shandong Agricultural University, Daizong Road, Tai’an 271018, China; 5 College of Horticulture, Agricultural University of Hebei, Baoding 071001, China

**Keywords:** transcriptome, senescence, darkness, bermudagrass

## Abstract

Leaf senescence induced by prolonged light deficiency is inevitable whenever turfgrass is cultivated in forests, and this negatively influences the survival and aesthetic quality of the turfgrass. However, the mechanism underlying dark-induced senescence in turfgrass remained obscure. In this study, RNA sequencing was performed to analyze how genes were regulated in response to dark-induced leaf senescence in bermudagrass. A total of 159,207 unigenes were obtained with a mean length of 948 bp. The differential expression analysis showed that a total of 59,062 genes, including 52,382 up-regulated genes and 6680 down-regulated genes were found to be differentially expressed between control leaves and senescent leaves induced by darkness. Subsequent bioinformatics analysis showed that these differentially expressed genes (DEGs) were mainly related to plant hormone (ethylene, abscisic acid, jasmonic acid, auxin, cytokinin, gibberellin, and brassinosteroid) signal transduction, N-glycan biosynthesis, and protein processing in the endoplasmic reticulum. In addition, transcription factors, such as WRKY, NAC, HSF, and bHLH families were also responsive to dark-induced leaf senescence in bermudagrass. Finally, qRT-PCR analysis of six randomly selected DEGs validated the accuracy of sequencing results. Taken together, our results provide basic information of how genes respond to darkness, and contribute to the understanding of comprehensive mechanisms of dark-induced leaf senescence in turfgrass.

## 1. Introduction

Cultivating turfgrass in forests has become a necessity of developing forestry economy and constructing gardens and landscapes. However, light deprivation caused by the forests exerts many negative effects on the turfgrass. When turfgrass is exposed to light-deficiency conditions, a series of morphological changes happen, including an increase in shoot elongation, leaf length, and leaf area [1,2,3]. Leaf senescence will be induced by prolonged and severe light deficiency. Leaf senescence is a critical concern for turfgrass because it negatively influences not only plant growth and development, but also the aesthetic turf quality. Therefore, it is important to investigate the mechanisms underlying dark-induced leaf senescence in order to develop ways to delay leaf senescence and improve turf quality under environments of light deficiency.

Leaf senescence is a complex and natural process at the final stage of leaf development, involving a series of changes in cellular physiology and biochemistry, enzyme activity, and gene expression [4]. Leaf senescence can as well be induced by abiotic stresses, such as darkness [5]. Previous studies have indicated that there is some overlap between natural senescence and dark-induced senescence [6]. Most of the genes that depend on ethylene and jasmonic acid (JA) for expression during natural leaf senescence also showed up-regulation during dark-induced senescence, indicating that these two pathways have an active signaling role in both age and dark-induced leaf senescence in *Arabidopsis* [6]. In rice plants, among 14 senescence-associated gene (SAG) clones characterized, 11 were found to be associated with both dark-induced and natural leaf senescence [7]. Several key age-dependent SAGs have been demonstrated to regulate dark-induced leaf senescence as well, such as *NON-YELLOW COLORING1/3* (*NYC1/3*), *EARLY FLOWERING3.1* (*ELF3.1*), *NAP*, *ORESARA1* (*ORE1*), and *DNA-binding with one finger 2.1* (*Dof2.1*) [8,9,10,11,12,13,14,15].

Despite all this, dark-induced and age-dependent senescence still triggers different gene expression profiles and signaling pathways [6,16,17]. Recent studies have demonstrated that the phytochrome-interacting factors (PIFs) signaling module plays a master role in dark-induced leaf senescence [18]. In *Arabidopsis*, *PIF4/PIF5* are central and necessary transcriptional activators of dark-induced leaf senescence. They are induced by darkness in a phytochrome B (phyB)-dependent manner. These two PIFs finally regulate chloroplast maintenance, hormone signaling, chlorophyll metabolism, and senescence master regulators during dark-induced senescence via multiple coherent feed-forward loops, together with *ORE1*, *ETHYLENE INSENSITIVE 3* (*EIN3*), and *ABA INSENSITIVE 5* (*ABI5*) and *ENHANCED EM LEVEL* (*EEL*) [19,20,21]. Light deprivation can result in a reduction in photosynthesis and subsequent carbon starvation. Some transcription factors involved in low-energy response also play important regulatory-roles in dark-induced leaf senescence. For example, overexpression of *BASIC LEUCINE ZIPPER63* (*bZIP63*) or *bZIP1* which were responsive to low energy could accelerate dark-induced leaf senescence [22,23].

Bermudagrass (*Cynodon dactylon* L.), frequently cultivated in sports fields, home lawns, and golf courses, is one of the most widely used warm-season turfgrass species [24]. However, dark-induced leaf senescence has not been studied yet in bermudagrass. Therefore, it is necessary to investigate the genome-wide response to dark stress, the most severe light-deficiency condition, in bermudagrass. RNA-sequencing is an important and direct approach to compare gene expression, and discover novel and rare transcripts in plants [25,26,27]. Identifying genes that are specifically responsive to dark-induced senescence by RNA-sequencing is the first and key step to explore the underlying mechanism of dark-induced senescence in bermudagrass. Therefore, in this study, prolonged dark treatment for the whole bermudagrass plants was used to initiate leaf senescence under laboratory conditions to mimic leaf senescence induced by light deficiency under natural conditions. Transcriptome analysis was then performed between senescent leaves induced by prolonging darkness and control leaves in bermudagrass. The objective of this study was to identify candidate genes and pathways involved in the dark-induced senescence process, and to provide basic information for elucidating the molecular mechanisms underlying dark-induced leaf senescence in bermudagrass.

## 2. Results

### 2.1. Dark Induced Leaf Senescence in Bermudagrass

**Figure 1:**
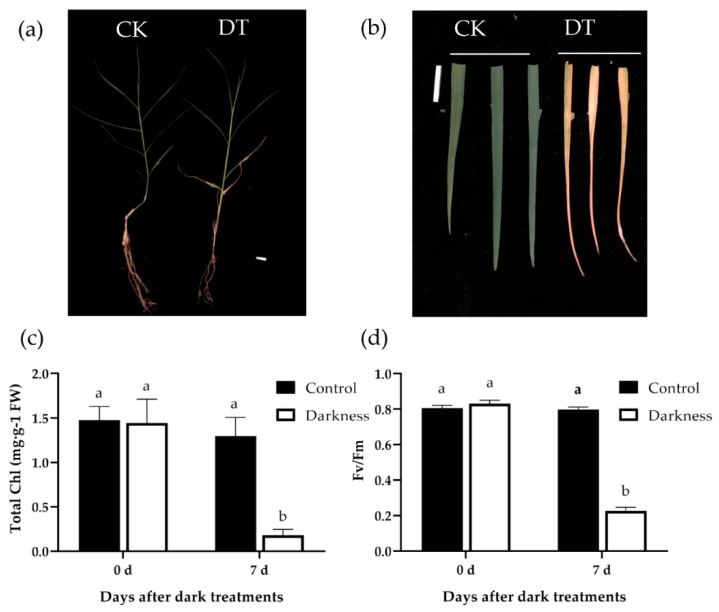
Phenotype analysis of bermudagrass after dark treatment. (**a**) Phenotype of whole plants after 7 d of control (left: CK) and dark (right: DT) treatments (scale = 1 cm); (**b**) phenotype of bottom leaves after 7 d of control (left: CK) and dark (right: DT) treatments (scale = 1 cm); (**c**,**d**) total chlorophyll contents (**c**) and Fv/Fm (**d**) of the first and second leaves from bermudagrass under control and dark treatments. Significant (*p* < 0.05) differences are indicated by different letters using Tukey’s HSD test. Error bars indicate the SD of three biological repeats. Fw—fresh weight.

To determine the effect of darkness on leaf senescence in bermudagrass, phenotypes of bermudagrass under dark treatment and control condition were examined. After 7 d of dark treatment (DT), leaf senescence initiated from the bottom, and severely yellowing leaves were observed in the bottom leaves (Figure 1a,b). However, all leaves of control plants (CK) still kept green. In addition, other senescence symptoms were also measured. Consistently, chlorophyll contents (Figure 1c) and PSII max-photo efficiencies (Fv/Fm) (Figure 1d) of the bottom leaves both declined after 7 d of dark treatment. However, there were no significant changes in bottom leaves in control plants after 7 d.

### 2.2. Characteristics of Genes in Response to Dark-Induced Leaf Senescence in Bermudagrass

To investigate genes responsive to dark-induced senescence in bermudagrass, a total of six cDNA libraries constructed from leaves with or without dark treatment (7 d) were sequenced using the Illumina Hiseq. After removing the low-quality reads and adaptors, 42,557,344; 53,558,496; 58,132,180; 64,587,208; 52,195,444, and 60,166,630 clean reads (>99%) were obtained for CK_1, CK_2, CK_3, DT_1, DT_2, and DT_3, respectively (Table 1). Subsequently, these high-quality clean reads were assembled using the Trinity de novo assembly program because of the absence of reference genome sequences. A total of 387,221 transcripts and 159,207 unigenes were obtained with a mean length of 1090 bp and 948 bp, respectively (Table 2). The results showed that sequencing quality was sufficiently high for downstream analyses.

### 2.3. Gene Annotation of the Bermudagrass Transcriptome

To analyze function of the 159,207 unigenes of bermudagrass, functional annotation of all unigenes was investigated using seven databases, including non-redundant protein sequence database (Nr), nucleotide sequence database (Nt), Pfam, clusters of orthologous groups of proteins (KOG/COG), Swiss-Prot, Kyoto encyclopedia of genes and genomes (KEGG), and gene ontology (GO). In total, there were 119,581 unigenes (75.11%) successfully annotated in at least one database, with 6961 unigenes (4.37 %) in all seven databases (Table 3). 

For GO analysis, genes involved in ‘metabolic process’ (46,108), ‘cellular process’ (47,512), and ‘single-organism process’ (38,511) were highly represented in the biological process. In terms of the cellular component category, ‘cell’ (24,404), ‘cell part’ (24,404), and ‘organelle’ (15,635) were highly enriched. Within the molecular function category, the major sub-categories were ‘binding’ (42,184), ‘catalytic activity’ (39,993), and ‘transporter activity’ (6464).

For KOG analysis, a total of 41,079 enriched unigenes were divided into 25 groups. The largest group was ‘posttranslational modification, protein turnover, chaperones’ (5483), followed by ‘general function prediction only’ (5130), and then ‘energy production and conversion’ (3949).

For KEGG analysis, 36,014 unigenes were classified into five main biochemical pathways: ‘cellular processes’, ‘environmental information processing’, ‘genetic information processing’, ‘metabolism’, and ‘organismal systems’. Within the metabolism pathway, ‘overview’, ‘carbohydrate metabolism’, and ‘amino acid metabolism’ were prominently represented. The pathways related to ‘genetic information processing’ included ‘folding, sorting, and degradation’, ‘replication and repair’, ‘transcription’, and ‘translation’. All function annotations provided important information for analyzing the processes, and pathways involved in dark-induced leaf senescence.

### 2.4. Differentially Expressed Genes (DEGs) in Response to Dark-Induced Leaf Senescence in Bermudagrass

**Figure 2:**
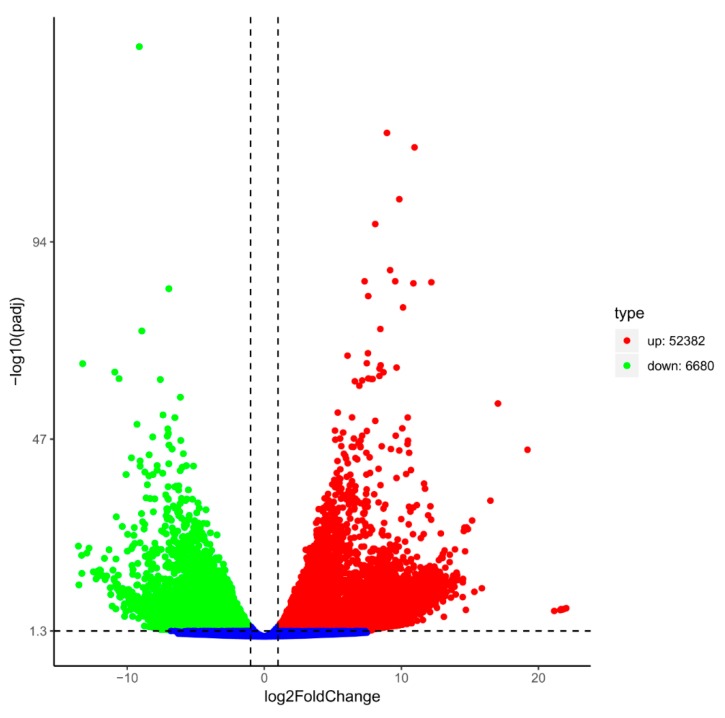
Volcano plot of transcriptional differences in Bermudagrass during dark-induced senescence. The vertical lines correspond to 2.0-fold up and down, and the horizontal line represents an adjusted *p*-value of 0.05. Red dots represent up-regulated differentially expressed genes (DEGs), and green dots represent down-regulated DEGs. DEGs, differentially expressed genes.

To investigate the global gene expressions in response to dark-induced leaf senescence, the differentially expressed genes between the control and dark-treated samples were analyzed. According to the criteria that at least a two-fold difference and an adjusted *p*-value less than 0.05 (|log2 (DT/CK)| ≥ 1, padj ≤ 0.05), a total of 59,062 genes, including 52,382 up-regulated genes and 6680 down-regulated genes, were found to be differentially expressed between CK and DT (Figure 2). It should be noted that the number of up-regulated genes was much larger than that of down-regulated genes, indicating that most of the genes were induced after dark treatment.

### 2.5. GO Analysis of Differentially Expressed Genes between CK and DT

**Figure 3:**
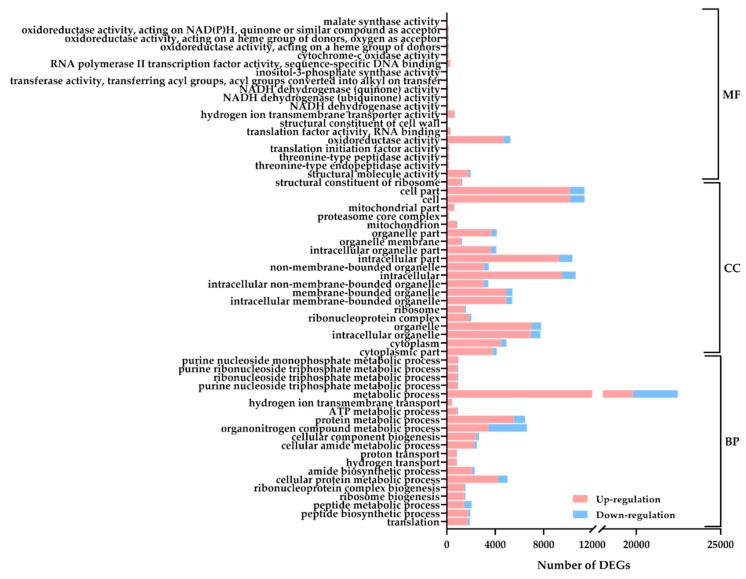
Gene Ontology (GO) classification of DEGs between CK and DT. DEGs were annotated in three categories: biological process (BP), cellular component (CC), and molecular function (MF). The enrichment index of each ontology term increased from the bottom up along Y-axis in all three categories.

To determine the biological processes of dark-responsive DEGs, GO analysis of DEGs between CK and DT was performed. A total of 32,383 DEGs could be assigned as a GO term and were annotated into 20 biological process items, 20 cellular component items, and 20 molecular function items (Figure 3, Table S1). Within the biological process category, translation, peptide biosynthetic process, and peptide metabolic process were the most enriched groups. Within the cellular component category, unigenes were prominently enriched in the cytoplasm part, cytoplasm, and intracellular organelle. In the molecular function category, unigenes were classified into a structural constituent of ribosome, structural molecule activity, and threonine-type endopeptidase activity. 

### 2.6. KEGG Pathway Enrichment Analysis of DEGs between CK and DT

To further explore the biological functions of the DEGs between CK and DT, KEGG pathway enrichment analysis was conducted (Figure S1). The up-regulated DEGs were annotated into 122 KEGG pathways. The significantly enriched pathways were ribosome, citrate cycle (TCA cycle), proteasome, oxidative phosphorylation, phagosome, protein processing in the endoplasmic reticulum, regulation of autophagy, endocytosis, pentose phosphate pathway, glycolysis/gluconeogenesis, and spliceosome. However, the down-regulated DEGs were involved in completely different pathways, such as photosynthesis, photosynthesis-antenna proteins, phenylpropanoid biosynthesis, plant hormone signal transduction, carotenoid biosynthesis, carbon fixation in photosynthetic organisms, fatty acid elongation, cyanoamino acid metabolism, and porphyrin and chlorophyll metabolism.

### 2.7. Genes Responsive to Dark-Induced Leaf Senescence in Bermudagrass

**Figure 4:**
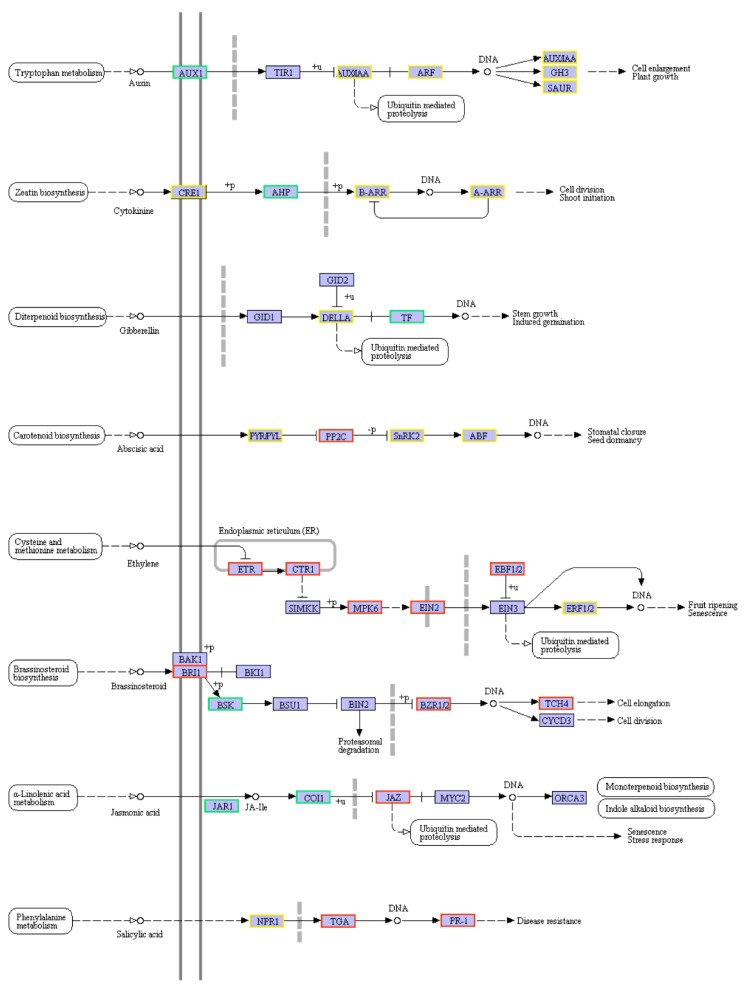
Differentially expressed genes between CK and DT in plant hormone signal transduction pathway. The red frame represents up-regulated genes, green frame represents down-regulated genes, and the yellow frame represents both up-regulated and down-regulated genes.

*Plant hormone signal transduction*. Plant hormones have been reported to play important roles either in natural leaf senescence or in dark-induced leaf senescence. Here, genes involved in plant hormone signal transduction pathways were differentially expressed during dark-induced leaf senescence in bermudagrass (Figure 4). Ethylene is thought to be a senescence-promoting phytohormone. Key components of the ethylene signal transduction pathway were almost all up-regulated after dark treatment, including six *ETHYLENE RESPONSES* (*ETR*s), one *CONSTITUTIVE TRIPLE RESPONSE 1* (*CTR1*), 17 MITOGEN-ACTIVATED PROTEIN KINASE 6 (*MPK6*), one *EIN2*, one *EIN3-BINDING F-BOX PROTEIN* 1/2 (EBF1/2), and one *ETHYLENE RESPONSE FACTOR 1/2* (*ERF1/2*). Only one *ERF* was down-regulated. Abscisic acid (ABA) level increases with dark-induced leaf senescence. DEGs involved in ABA signal transduction were also highly represented and most unigenes were up-regulated as well. The expression of one *PYL*, 11 TYPE 2C PROTEIN PHOSPHATASES (*PP2C*s), nine *SUCROSE NONFERMENTING 1-RELATED PROTEIN KINASE 2S* (*SnRK2s*), and two *ABA-RESPONSIVE ELEMENT-BINDING FACTORS* (*ABF*s) were up-regulated during dark-induced leaf senescence. For salicylic acid, there was one up-regulated *PATHOGENESIS-RELATED* GENES 1 (*NPR1*), ten up-regulated *TGACG SEQUENCE-SPECIFIC BINDING PROTEINS* (*TGA*s), ten up-regulated *PATHOGENESIS-RELATED PROTEIN 1s* (*PR-1s*), and one down-regulated *NPR1* after dark treatment. JA is critically involved in senescence. Application of JA induces premature senescence and the level of JA increases with leaf senescing. During the dark-induced leaf senescence, several genes involved in JA signaling or response were differentially regulated. *JASMONATE RESISTANT* (*JAR*) and CORONATINE-INSENSITIVE PROTEIN 1 (*COI1*) genes were both down-regulated. As the direct target of *COI1*, all *JASMONATE ZIM-DOMAIN* (*JAZ*) genes were up-regulated as expected. Exogenous application or endogenous overexpression of cytokinin can delay leaf senescence. Although no genes involved in cytokinin biosynthesis were identified with differential expression, several genes involved in cytokinin signaling pathway (A multistep ARABIDOPSIS HISTIDINE KINASE (AHK) → ARABIDOPSIS HISTIDINE-CONTAINING PHOSPHOTRANSMITTER (AHP) → ARABIDOPSIS THALIANA RESPONSE REGULATORS (ARR) phosphorelay pathway) were up- or down-regulated during dark-induced senescence. Only one *AHK4* and one *AHP* were detected with down-regulation. One A-type *ARR* genes and two B-type *ARR* genes were suppressed, whereas three A-type *ARR* genes and one B-type *ARR* genes were induced. The role of auxin in leaf senescence is still controversial. AUXIN RESPONSE FACTOR (ARF) and AUXIN/INDOLE-3-ACETIC ACID (Aux/IAA) proteins are crucial factors in the auxin response pathway. Expression of almost all *ARF* genes was decreased in the senescent leaves induced by dark except for that of *ARF16*. Similar to the *ARFs*, 3/4 of the *Aux/IAA* genes are down-regulated during dark-induced senescence. In addition, genes participating in signal transduction of gibberellin, and brassinosteroid were also regulated during dark-induced senescence (Table S2).

**Figure 5:**
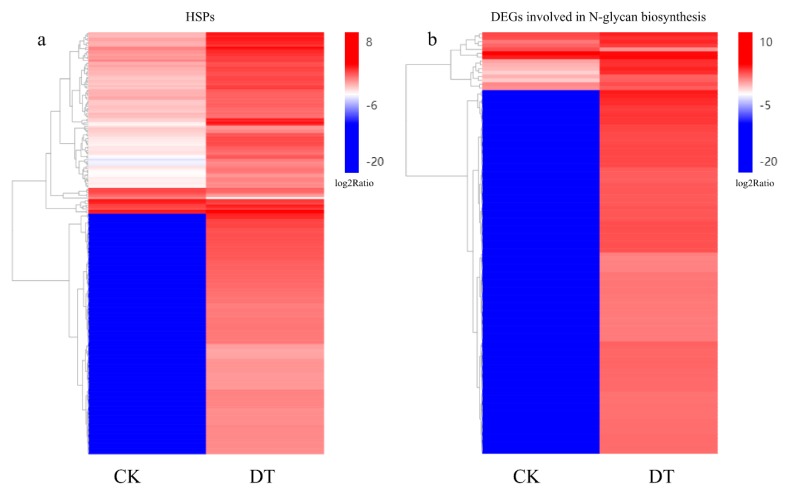
Hierarchical clustering analysis of DEGs between CK and DT. (**a**) Heat shock proteins (*Hsps*) were largely and significantly induced by dark-induced leaf senescence; (**b**) Almost all DEGs involved in N-glycan biosynthesis were activated during dark-induced leaf senescence. Different color bands represent the different expression level of each sample. The red bands represent a high expression level and the blue bands represent low expression levels.

*Protein processing in the endoplasmic reticulum.* Up-regulated DEGs were significantly enriched in protein processing in the endoplasmic reticulum pathway. Among genes involved in protein processing in the endoplasmic reticulum, one of the most conspicuous changes was the up-regulation of *heat shock proteins* (*Hsps*). Hsps were reported to play key roles in aging phenotypes in animals [28]. However, little is known about their roles in leaf senescence, especially dark-induced leaf senescence. During dark treatment, a total of 221 *Hsps* were up-regulated, including *Hsp20s, Hsp70s,* and *Hsp90s* (Figure 5a). Only nine *Hsps* were suppressed after 7 d of dark treatment.

*N-glycan biosynthesis.* It is worth noting that dark-induced leaf senescence activated almost all genes participating in N-glycan biosynthesis. A total of 109 DEGs were involved in N-glycan biosynthesis (Figure 5b), 107 of which were up-regulated, including *ASPARAGINE-LINKED GLYCOSYLATION1,2,3,4,6,7,9,11,13,14* (*ALG1*,*2*,*3*,*5*,*6*,*7*,*9*,*11*,*13*,*14*), *DOLICHOL-PHOSPHATE MANNOSYLTRANSFERASE 1,2,3* (*DPM1*,*2*,*3*), *STAUROSPORIN AND TEMPERATURE SENSITIVE* (*STT*), *OLIGOSACCHARYLTRANSFERASE* (*OST*), *MANNOSYL-OLIGOSACCHARIDE GLUCOSIDASE 1* (*GCS1*), *MANNOSYL-OLIGOSACCHARIDE ALPHA-1,2-MANNOSIDASE* (*MAN1*), *MANNOSYL-OLIGOSACCHARIDE ALPHA-1,3-GLUCOSIDASE* (*GANAB*), and *ALPHA-1,6-MANNOSYL-GLYCOPROTEIN BETA-1,2-N-ACETYLGLUCOSAMINYLTRANSFERASE* (*MGAT2*). Only one *OST* and one *MGAT3* were down-regulated during dark-induced senescence. 

### 2.8. Regulation of Transcription Factor (TF) Families during Dark-Induced Leaf Senescence

NAC and WRKY are two of the most important transcription factor families in the regulation of leaf senescence [8,9,29,30,31,32,33]. In this study, members of various NAC (Table S3) and WRKY TFs (Table S4) were identified during dark-induced senescence. Overall, the number of up-regulated WRKY and NAC TFs was greater than that of down-regulated ones. Of the DEGs encoding NAC and WRKY TFs, 40 and 25 were up-regulated, while only 5 and 10 were down-regulated respectively. PIFs, a group of basic helix-loop-helix (bHLH) family, have been demonstrated as major transcription factors orchestrating dark-induced senescence [18]. A total of three *PIFs* were identified with significantly different expressions, including two *PIF4s* and one *PIF3* (Table 4). The expression levels of these three *PIFs* were all suppressed in dark-induced senescent leaves. To investigate the expression pattern of *CdPIF3/4* during dark-induced senescence, expression levels of one *PIF3* and one *PIF4* were further analyzed by quantitative real-time PCR (qRT-PCR) after 0, 1, 2, 3, 5, and 7 d of dark treatment (Figure S2). Both *CdPIF3* and *CdPIF4* were highly induced after the first five days’ dark treatment, but reduced after 7 d of dark treatment. In addition, corresponding to the numerous *Hsps* which were up-regulated in senescent leaves induced by darkness, a total of six *heat shock transcription factors* (*Hsfs*) with up-regulated expression were also identified (Table S5). 

### 2.9. Validating the DEGs by qRT-PCR Analysis

**Figure 6:**
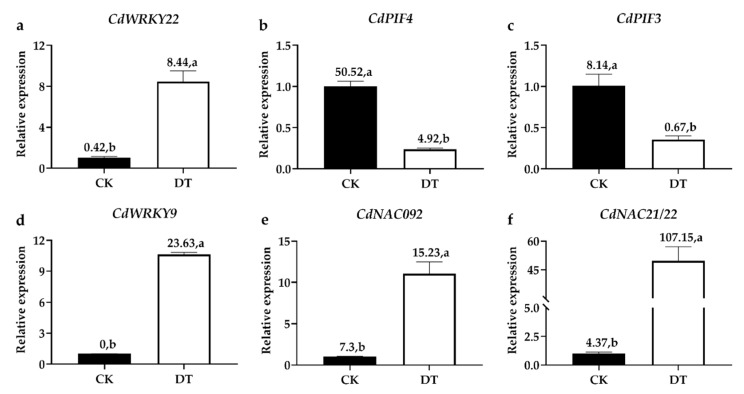
Real-Time RT-PCR (qRT-PCR) validation of DEGs in response to dark-induced leaf senescence. Expression analysis of (**a**) *CdWRKY22*; (**b**) *CdPIF4*; (**c**) *CdPIF3*; (**d**) *CdWRKY9*; (**e**) *CdNAC092*; (**f**) *CdNAC21/22* between CK and DT by qRT-PCR. The numbers above the error bars represent the fragments per kilobase of transcript per million fragments mapped (FPKM) obtained by RNA-sequence in CK and DT. Letters above error bars indicate a significant difference (*p* < 0.05) between CK and DT using Student’s t-test. The error bars represent the SD value (*n* = 3).

To validate the results of RNA sequencing, six DEGs including *CdPIF3*, *CdPIF4*, *CdNAC092*, *CdWRKY9*, *CdWRKY22*, and *CdNAC21/22* were randomly selected for qRT-PCR. The qRT-PCR results were basically consistent with those of RNA sequencing, suggesting that the RNA sequencing results were reliable (Figure 6). 

## 3. Discussion

In this study, RNA sequencing was performed in CK and DT of bermudagrass. Our results suggested that dark responsive genes were mainly involved in plant hormone signal transduction, N-glycan biosynthesis, and protein processing in the endoplasmic reticulum. In addition, NAC, WRKY, bHLH, and HSF TF families were also responsive to dark-induced leaf senescence in bermudagrass. 

PIFs have been demonstrated as master transcription factors during dark-induced leaf senescence [18]. Among the seven PIFs in *Arabidopsis*, PIF3, PIF4, and PIF5 function as the central and essential transcriptional activators of dark-induced senescence [19,20]. Their transcriptional and protein levels were both induced by darkness. In this study, significantly different expressions of three *PIFs* including one *PIF3* and two *PIF4s* were detected between CK and DT in bermudagrass (Table 4). However, their expression levels were all suppressed by 7 d of dark treatment. This was probably because *PIF3*, *4*, and *5* are early senescence responsive genes. Their expression levels were induced at the first two days of dark treatment but reduced at day 3 post-treatment in *Arabidopsis* [20]. In *Solanum lycopersicum*, *SlPIF4* also exhibited expression tendency similar to that reported for *A. thaliana* homologs during dark-induced senescence [34]. Further qRT-PCR results demonstrated that the expression levels of *CdPIF3/PIF4* were indeed induced by short-time darkness and then declined after long-time darkness in bermudagrass, indicating that the *CdPIF3/4* are early dark-induced senescence responsive genes (Figure S2). 

PIFs were reported to regulate dark-induced leaf senescence by targeting hormone signaling and production. They were shown to promote dark-induced senescence by inducing both ethylene biosynthesis and signaling, and ABA signal transduction via *1-aminocyclopropane-1-carboxylate synthases* (*ACSs*), *EIN3, EEL,* and *ABI5* in *Arabidopsis* [19,35,36,37,38]. In contrast, the expression levels of *CdACS*, *CdEIN3*, *CdEEL,* and *CdABI5* were not up- or down-regulated during dark-induced senescence in bermudagrass. However, other genes involved in ethylene and ABA signaling were identified. For example, *EIN2*, a positive regulator of aging-induced leaf senescence [39,40,41], was largely induced, although *EIN2* was not detected on the array of senescent leaves induced by dark treatment in *Arabidopsis* [42]. In addition to ethylene and ABA, other plant hormones, including JA, SA, cytokinin, auxin, gibberellin, and brassinosteroid, which have not been reported to be targeted by PIFs also participated in dark-induced leaf senescence in bermudagrass. Cytokinin can delay leaf senescence and the level of cytokinin is reduced in senescent leaves. Here, most of *CdAHK4s*, cytokinin receptors were induced except one *CdAHK4*. In the cytokinin AHK→AHP→ARR phosphorelay pathway, A-type *ARRs* are reported to be rapidly up-regulated by cytokinin [43]. Due to the decline of cytokinin during senescence, *ARRs* were observed to be down-regulated in the previous studies [6,42]. In contrast, three of four *ARRs* were significantly induced in this study, suggesting that they may have a special function of cytokinin signaling during dark-induced leaf senescence in bermudagrass. A similar phenomenon happened to *AtARR16*, which was also up-regulated during dark-induced leaf senescence in *Arabidopsis* [42]. JA plays a pivotal role in dark-induced leaf senescence. *JAZ* genes were found to be significantly induced under dark treatment, and several *JAZ* genes, such as *JAZ7*, were reported to negatively regulate dark-induced leaf senescence in *Arabidopsis* [44,45]. Consistently, all *JAZ* genes were induced during dark-induced leaf senescence in bermudagrass. In addition, the SA signaling pathway was also enriched in dark-induced senescence in bermudagrass, although it was not detected in *Arabidopsis* [6].

Many transcription factors have been identified as master senescence regulators. During dark-induced leaf senescence, the senescence key regulator *AtNAC092* is activated by *PIF3/4/5*, *EIN3*, *ABI5,* and *EEL* [19]. In our study, we found *CdNAC092* was significantly induced although the expression levels of *CdPIF3/4* were down-regulated. Another two senescence master regulators *WRKY22* and *NAP* are also affected by PIFs directly or indirectly in *Arabidopsis* during dark-induced leaf senescence [8,29,36]. Here, *CdWRKY22* and *CdNAC029* were significantly induced under dark-induced leaf senescence as well. These results suggest that the senescence master regulators might play roles during dark-induced senescence through additional PIF-independent ways. 

It has been reported that N-glycan alteration is associated with age in humans [46]. In plants, N-glycans were reported to play an important role during fruit ripening. Two ripening-specific N-glycoprotein modifying enzymes, α-mannosidase (α-Man) and β-D-N-acetylhexosaminidase (β-Hex) have been identified [47]. However, the role of N-glycan in leaf aging, especially dark-induced leaf senescence is unclear. In this study, genes involved in N-glycan biosynthesis were almost all significantly induced during dark-induced leaf senescence in bermudagrass, indicating that N-glycan might be associated with dark-induced leaf senescence in bermudagrass.

HSPs are molecular chaperones and have been implicated in longevity and aging in many species, such as *Caenorhabditis elegans* [28]. However, their association with leaf aging, especially dark-induced leaf senescence is unknown. In this study, it is interesting to note that numerous *Hsps* were induced during dark-induced leaf senescence, indicating that *Hsps* may refold the damaged proteins accumulating during dark-induced leaf senescence to maintain protein homeostasis and longevity. As we know, the expression of *Hsp* genes is controlled by *Hsfs*. As expected, several Hsfs were indeed up-regulated in the senescent leaves induced by darkness here. It was reported that overexpression of *HaHsf9* resulted in a slowing of dark-induced seedling senescence [48]. The function of *CdHsfs* in dark-induced leaf senescence is yet to be revealed.

## 4. Conclusions

**Figure 7:**
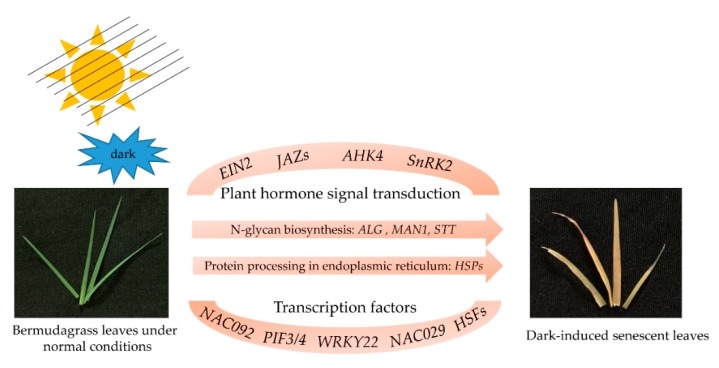
The mode pattern of dark/shade-induced leaf senescence in bermudagrass.

In this study, to better understand the mechanism of dark-induced leaf senescence in bermudagrass, the comprehensive differences in transcription levels between CK and DT in bermudagrass were investigated by RNA-sequencing. A total of 59,062 DEGs, including 52,382 up-regulated and 6680 down-regulated genes were identified as being significantly responsive to dark-induced leaf senescence. Bioinformatics analysis (Figure 7) revealed that these DEGs were mainly associated with plant hormone signal transduction (*CdEIN2*, *CdSnRK2*, *CdJAZs*, *CdAHK4*), N-glycan biosynthesis (*CdALG*, *CdSTT*, *CdMAN1*), and protein processing in the endoplasmic reticulum (*CdHsps*). Several key transcription factors (*Cd*PIF3/4, CdWRKY22, CdNAC029, and CdNAC092) were also identified during dark-induced leaf senescence. This study provides important information for the identification of genes involved in dark-induced leaf senescence in bermudagrass. Further function analyses of these candidate genes are required to reveal the molecular mechanisms underlying this complex and highly coordinated developmental process.

## 5. Materials and Methods

### 5.1. Plant Materials and Treatments 

The bermudagrass used in this study was collected from a wild field, Zhejiang province, China. To ensure the same genetic background, stolons collected from one plant were planted in plastic pots (15 cm diameter × 20 cm tall) filled with matrix (brown coal soil:sand = 1:1) to establish the seedlings. The plants were cultivated in a controlled-environment growth chamber (HP300GS-C, Ruihua Instrument, Wuhan, China), with a 16 h photoperiod, photosynthetically active radiation at 450 μmol m^−2^ s^−1^ at the canopy level, a day/night temperature of 28/24°C and 70% relative humidity. After two months of establishment, the fully developed bermudagrass plants were divided into two groups. Group I was maintained in the same chamber with the same growth conditions. Group II was transferred to another controlled-environment growth chamber with the same temperature and humidity but no light. One week later, green leaves were collected 4 h after the beginning of the light period from Group I (named as CK), and dark-induced senescent leaves were collected from Group II (named as DT). Leaf samples were collected and frozen immediately with liquid nitrogen, and stored at −80 °C for future analysis. All the treatments were repeated for three replications.

### 5.2. RNA Preparation

The total RNA of bermudagrass leaf was extracted by Trizol reagent (Invitrogen, Carlsbad, CA, USA) according to the manufacturer’s protocol. The NanoPhotometer^®^ spectrophotometer (IMPLEN, Westlake Village, CA, USA) was applied to check the RNA purity. To guarantee the RNA purity, degradation, and contamination of RNA were monitored on 1% agarose gels. The Qubit^®^ RNA Assay Kit was applied to measure the RNA concentration in Qubit^®^ 2.0 Fluorometer (Life Technologies, Carlsbad, CA, USA). The RNA Nano 6000 Assay Kit of the Agilent Bioanalyzer 2100 system (Agilent Technologies, Santa Clara, CA, USA) was applied to detect the RNA integrity.

### 5.3. Library Preparation for Transcription Sequencing

A total of 1.5 μg RNA per sample was used for library preparation. The sequencing libraries were generated by NEBNext^®^ Ultra™ RNA Library Prep Kit for Illumina^®^ (NEB, Ipswich, MA, USA) according to the manufacturer’s recommendations. Simultaneously, the index codes were added for attributing sequences to each sample. In brief, after mRNA purification from total RNA with poly-T oligo-attached magnetic beads, the fragmentation was carried out under elevated temperature by divalent cations in NEBNext First Strand Synthesis Reaction Buffer. Then random hexamer primer and M-MuLV Reverse Transcriptase (RNase H^-^) was applied to the synthesized first-strand cDNA, and DNA polymerase I and RNase H were used to obtain the second strand DNA. The remaining overhangs were converted to blunt ends by exonuclease/polymerase. The NEBNext Adaptor with a hairpin loop structure was ligated to adenylated 3′ ends of DNA fragments to prepare for hybridization reaction. During this process, the fragments were purified by AMPure XP system (Beckman Coulter, Beverly, CA, USA) to select the cDNA fragments in length of 250–300 bp. Before PCR, 3 µL USER Enzyme (NEB, Ipswich, MA, USA) was applied with size-selected, adaptor-ligated cDNA for 15 min at 37 °C followed by 5 min at 95 °C. To perform PCR, Universal PCR primers, Index (X) Primer, and Phusion High-Fidelity DNA polymerase were used. The PCR products were purified with AMPure XP system and the library quality was estimated with Agilent Bioanalyzer 2100 system.

The clustering of the index-coded samples was conducted by the cBot Cluster Generation System with the application of TruSeq PE Cluster Kit v3-cBot-HS (Illumina, San Diego, CA, USA). After that, the samples were sequenced on an Illumina Hiseq platform.

### 5.4. Data Analysis

#### 5.4.1. Quality Control

To obtain the clean data, the reads containing adapter and ploy-N as well as low-quality reads were removed from the raw data. Simultaneously, Q20, Q30, GC-content and sequence duplication levels of the clean data were calculated. The high-quality clean data was used for downstream analyses.

#### 5.4.2. Transcriptome Assembly

Transcriptome assembly was performed with Trinity [49], min_kmer_cov was set to 2 by default and other parameters were set to default.

#### 5.4.3. Gene Functional Annotation

Gene functions were annotated based on the databases of Nr (NCBI nonredundant protein sequences, ftp://ftp.ncbi.nih.gov/blast/db), Nt (NCBI nonredundant nucleotide sequences, ftp://ftp.ncbi.nih.gov/blast/db), Pfam (Protein family, http://pfam.sanger.ac.uk/), KOG/COG (Clusters of Orthologous Groups of proteins, http://www.ncbi.nlm.nih.gov/COG/), Swiss-Prot (A manually annotated and reviewed protein sequence database, http://www.ebi.ac.uk/uniprot/), KO (KEGG Ortholog database, http://www.genome.jp/kegg/) and GO (Gene Ontology, http://www.geneontology.org/).

#### 5.4.4. Differential Expression Analysis

Differential expression analysis was performed by the DESeq R package (1.10.1). To control the false discovery rate, the Benjamini and Hochberg’s approach was applied to adjust the resulting *p* values. Genes with an adjusted *p*-value < 0.05 were assigned as differentially expressed genes (DEGs).

#### 5.4.5. GO Enrichment Analysis

Gene Ontology (GO) enrichment analysis of the DEGs was conducted by the GOseq R packages which were based on Wallenius noncentral hypergeometric distribution [50].

#### 5.4.6. KEGG Pathway Enrichment Analysis

For KEGG pathway enrichment analysis, KOBAS software was applied to test the statistical enrichment of DEGs in KEGG pathways [51].

### 5.5. Validation of RNA-Sequence Data by Quantitative Real-Time PCR (qRT-PCR)

The expression levels of DEGs were performed by qRT-PCR following the previously described method [52]. In brief, cDNA synthesis was performed using a PrimeScript RT reagent kit (Takara, Dalian, China). The qRT-PCR was performed using 20 μL volumes of SYBR (Takara, Dalian, China) and the ABI STEPONE Real-Time PCR system. The thermal cycle parameters used were as follows: 30 s at 95 °C, 40 cycles of 10 s at 95 °C, and 30 s at 60 °C. To confirm that only one PCR product was amplified and detected, a melting curve analysis of amplification products was performed at the end of each PCR reaction. *CdACTIN2* was selected as a reference gene for normalization. All gene-specific primers used for qRT-PCR analysis are listed in Table S6. Three independent biological replicates were used for qRT-PCR analysis.

### 5.6. Measurements of Chlorophyll Content and Fv/Fm

The leaves were cut into small pieces and incubated in 5 mL dimethylsulfoxide for 12 h under dark conditions for chlorophyll extraction. Chlorophyll was detected by measuring absorption (OD) at 649 and 665 nm with a spectrophotometer (UV-2600, UNICO, Dayton, NJ, USA), and chlorophyll content (mg·g^−1^ FW) was calculated as (18.08 × OD649 + 6.63 × OD665) × 0.005/W.

Before measuring Fv/Fm, leaves were kept in the dark for 30 min to close all PSII reaction centers. Then Fv/Fm was measured by a pulse-amplitude modulation (PAM) portable chlorophyll fluorometer (PAM- 2500, WALZ, Effeltrich, Germany) according to the manufacturer’s protocol.

## Figures and Tables

**Table 1:** Characteristics of RNA-sequencing results of all samples.

Sample	Raw Reads	Clean Reads	Clean Bases	Error (%)	Q20(%) ^1^	Q30(%) ^2^	GC(%) ^3^
CK_1	45,224,598	42,557,344	6.38	0.03	97.90	94.07	51.53
CK_2	55,052,156	53,558,496	8.03	0.02	98.21	94.71	53.28
CK_3	59,896,392	58,132,180	8.72	0.02	98.35	95.04	53.44
DT_1	67,686,056	64,587,208	9.69	0.02	98.03	94.40	52.53
DT_2	54,946,418	52,195,444	7.83	0.02	98.10	94.51	52.67
DT_3	65,845,158	60,166,630	9.02	0.02	98.00	94.31	52.34

^1^ Q20 (%) is the proportion of nucleotides with a quality value larger than 20. ^2^ Q30 (%) is the proportion of nucleotides with a quality value larger than 30. ^3^ GC (%) is the proportion of guanidine and cytosine nucleotides among the total nucleotides.

**Table 2:** The number of the transcripts and unigenes with different kinds of length clustered from the de novo assembly.

Category	Transcripts	Unigenes
200–500 bp	128,446	60,507
500–1 kbp	111,291	53,622
1–2 kbp	97,001	30,181
>2 kbp	50,483	14,897
Min length (bp) ^1^	301	301
Mean length (bp)	1090	948
Max length (bp) ^2^	53,494	53,494
N50 length (bp) ^3^	1559	1284
N90 length (bp) ^4^	465	426
Total	387,221	159,207

^1^ The Min length means the minimum length of transcripts or unigenes. ^2^ The Max length means the maximum length of transcripts and unigenes ^3^ The N50 value is defined as the contig length where half of the assembly is represented by contigs of this size or longer. ^4^ The N90 value is defined as the contig length where 90% of the assembly is represented by contigs of this size or longer.

**Table 3:** The numbers and distribution rate of unigenes in the databases of the non-redundant protein sequence (Nr), nucleotide sequence (Nt), Kyoto encyclopedia of genes and genomes (KEGG), Swiss-Prot, Pfam, gene ontology (GO), and clusters of orthologous groups of proteins (KOG/COG).

Categories	Number of Unigenes	Percentage (%)
Annotated in NR	39,336	24.7
Annotated in NT	76,881	48.28
Annotated in KEGG	36,014	22.62
Annotated in Swiss-Prot	83,705	52.57
Annotated in PFAM	84,295	52.94
Annotated in GO	84,295	52.94
Annotated in KOG	41,079	25.8
Annotated in all Databases	6961	4.37
Annotated in at least one Database	119,581	75.11
Total Unigenes	159,207	100

Nr, non-redundant protein sequence database; Nt, nucleotide sequence database; KEGG, Kyoto encyclopedia of genes and genomes; KOG, clusters of orthologous groups of proteins; GO, gene ontology.

**Table 4:** The differentially expressed phytochrome-interacting factors (PIFs) transcription factors (TFs) between CK and DT.

GeneID	Log_2_(DT/CK)	padj-value	Gene Description
Cluster-109720.12769-2R	−2.8599	1.71 × 10^−16^	Transcription factor PIF4 OS = *Arabidopsis thaliana* GN = PIF4 PE = 1 SV = 1
Cluster-107104.0-1F	−7.2349	1.02 × 10^−19^	Transcription factor PIF4 OS = *Arabidopsis thaliana* GN = PIF1 PE = 1 SV = 1
Cluster-113186.0-2F	−5.49	1.41 × 10^−10^	Transcription factor PIF3 OS = *Oryza sativa* subsp. japonica GN = PIF3 PE = 1 SV = 1

## Supplementary Materials

The following are available online at https://www.mdpi.com/2223-7747/8/12/614/s1. Table S1. Gene Ontology (GO) classification of DEGs between CK and DT. Table S2. DEGs involved in signal transduction of gibberellin, and brassinosteroid. Table S3. The differentially expressed NAC TFs between CK and DT. Table S4. The differentially expressed WRKY TFs between CK and DT. Table S5. The differentially expressed HSF TFs between CK and DT. Table S6. All primers used in this study. Figure S1. Kyoto encyclopedia of genes and genomes (KEGG) analysis of DEGs between CK and DT. (a) KEGG analysis of up-regulated DEGs; (b) KEGG analysis of down-regulated DEGs. Figure S2. qRT-PCR analysis of CdPIF3 and CdPIF4 during dark-induced leaf senescence. Relative expression of (a) CdPIF3; (b) CdPIF4 in the first and second leaves from bermudagrass after 1, 1, 2, 3, 5 and 7 d of dark treatments. Letters above error bars indicate significant difference (*p* < 0.05) using Tukey’s HSD test. The error bars represent the SD value (*n* = 3).
