# Supplementary material for: Transcriptomic Analysis of Dark-Induced Senescence in Bermudagrass (Cynodon dactylon)
Source: Plants (Basel). 2019 Dec 17;8(12):614. doi: 10.3390/plants8120614 (PMC6963411; doi:10.3390/plants8120614)
Supplement: Supplementary file 1 [file plants-08-00614-s001.pdf]

plants

MDPI

Table S1. Gene Ontology (GO) classification of DEGs between CK and DT.

	GO_Term	Ontology	Number	Corrected *p*-value
BP	GO:0006412	translation	1887	3.27E−27
	GO:0043043	peptide biosynthetic process	1923	4.25E−26
	GO:0006518	peptide metabolic process	2047	7.15E−25
	GO:0042254	ribosome biogenesis	1529	7.15E−25
	GO:0022613	ribonucleoprotein complex biogenesis	1543	1.98E−24
	GO:0044267	cellular protein metabolic process	5018	8.53E−21
	GO:0043604	amide biosynthetic process	2297	1.24E−18
	GO:0006818	hydrogen transport	850	4.19E−17
	GO:0015992	proton transport	850	4.19E−17
	GO:0043603	cellular amide metabolic process	2500	2.67E−16
	GO:0044085	cellular component biogenesis	2662	1.74E−15
	GO:1901564	organonitrogen compound metabolic process	6610	5.03E−14
	GO:0019538	protein metabolic process	6457	8.58E−12
	GO:0046034	ATP metabolic process	929	4.15E−11
	GO:1902600	hydrogen ion transmembrane transport	454	7.67E−11
	GO:0008152	metabolic process	22445	1.89E−10
	GO:0009144	purine nucleoside triphosphate metabolic process	939	2.17E−10
	GO:0009199	ribonucleoside triphosphate metabolic process	939	2.17E−10
	GO:0009205	purine ribonucleoside triphosphate metabolic process	939	2.17E−10
	GO:0009126	purine nucleoside monophosphate metabolic process	956	2.86E−10
CC	GO:0044444	cytoplasmic part	4116	4.38E−49
	GO:0005737	cytoplasm	4937	1.65E−47
	GO:0043229	intracellular organelle	7726	2.31E−47
	GO:0043226	organelle	7793	9.95E−45
	GO:0030529	ribonucleoprotein complex	2020	3.78E−44
	GO:0005840	ribosome	1589	3.21E−42
	GO:0043231	intracellular membrane-bounded organelle	5406	1.98E−24
	GO:0043227	membrane-bounded organelle	5415	1.98E−24
	GO:0043232	intracellular non-membrane-bounded organelle	3416	5.62E−20
	GO:0005622	intracellular cellular_component	10631	5.95E−18
	GO:0043228	non-membrane-bounded organelle	3457	6.58E−18
	GO:0044424	intracellular part	10371	7.51E−17
	GO:0044446	intracellular organelle part	4091	3.13E−13
	GO:0031090	organelle membrane	1265	6.80E−13
	GO:0044422	organelle part	4118	1.80E−12
	GO:0005739	mitochondrion	860	2.12E−12
	GO:0005839	proteasome core complex	180	1.95E−11
	GO:0044429	mitochondrial part	637	4.15E−11
	GO:0005623	cell	11369	4.34E−11
	GO:0044464	cell part	11369	4.34E−11
MF	GO:0003735	structural constituent of ribosome	1270	2.82E−35
	GO:0005198	structural molecule activity	1966	2.07E−17
	GO:0004298	threonine-type endopeptidase activity	166	5.23E−12
	GO:0070003	threonine-type peptidase activity	166	5.23E−12
	GO:0003743	translation initiation factor activity	216	3.06E−08
	GO:0016491	oxidoreductase activity	5248	2.57E−07
	GO:0008135	translation factor activity, RNA binding	314	3.82E−07
	GO:0005199	structural constituent of cell wall	56	6.78E−07
	GO:0015078	hydrogen ion transmembrane transporter activity	655	8.42E−07
	GO:0003954	NADH dehydrogenase activity	135	6.13E−06

GO:0008137	NADH dehydrogenase (ubiquinone) activity	125	8.07E−06
GO:0050136	NADH dehydrogenase (quinone) activity	125	8.07E−06
GO:0046912	transferase activity, transferring acyl groups, acyl groups converted into alkyl on transfer	112	1.34E−05
GO:0004512	inositol-3-phosphate synthase activity	28	6.75E−05
GO:0000981	RNA polymerase II transcription factor activity, sequence-specific DNA binding	297	0.00017828
GO:0004129	cytochrome-c oxidase activity	158	0.00049417
GO:0016675	oxidoreductase activity, acting on a heme group of donors	158	0.00049417
GO:0016676	oxidoreductase activity, acting on a heme group of donors, oxygen as acceptor	158	0.00037592
GO:0016655	oxidoreductase activity, acting on NAD(P)H, quinone or similar compound as acceptor	156	0.00049417
GO:0004474	malate synthase activity	46	0.00052106

Table S2. DEGs involved in signal transduction of gibberellin, and brassinosteroid.

GeneID	Log2(DT/CK)	padj -value	Gene Description
Cluster-97206.1	3.3794	9.68E−05	DELLA protein DWARF8 OS = Zea mays GN = D8 PE = 1 SV = 1
Cluster-86642.0	3.0942	4.62E−08	DELLA protein GAI OS = Arabidopsis thaliana GN = GAI PE = 1 SV = 1
Cluster-109720.10441	−1.3811	0.000718	DELLA protein DWARF8 OS = Zea mays GN = D8 PE = 1 SV = 1
Cluster-109720.11060	−7.2349	1.02E−19	Transcription factor PIF1 OS = Arabidopsis thaliana GN = PIF1 PE = 1 SV = 1
Cluster-109720.12769	−2.8599	1.71E−16	Transcription factor PIF4 OS = Arabidopsis thaliana GN = PIF4 PE = 1 SV = 1
Cluster-109720.22043	−5.49	1.41E−10	Transcription factor APG OS = Oryza sativa subsp. japonica GN = APG PE = 1 SV = 1
Cluster-78574.0	1.9032	1.74E−05	Systemin receptor SR160 OS = Solanum peruvianum PE = 1 SV = 1
Cluster-109720.23586	−1.6167	0.02647	Probable serine/threonine-protein kinase At5g41260 OS = Arabidopsis thaliana GN = At5g41260 PE = 1SV = 1
Cluster-109720.3941	1.2357	0.004413	Protein BZR1 homolog 1 OS = Oryza sativa subsp. indica GN = BZR1 PE = 3 SV = 1
Cluster-109007.0	2.2975	0.001821	Xyloglucan endotransglucosylase/hydrolase protein 22 OS = Arabidopsis thaliana GN = XTH22 PE = 1 SV = 1
Cluster-107056.0	1.9144	7.79E−05	Probable xyloglucan endotransglucosylase/hydrolase protein 23 OS = Arabidopsis thaliana GN = XTH23 PE = 2 SV = 1

Table S3. The differentially expressed NAC TFs between CK and DT.

GeneID	Log_2(DT/CK)	q-value	Gene Description
Cluster-109720.274-0R	3.3094	0.001291	NAC transcription factor NAM-A1 OS = Triticum turgidum subsp. durum GN = NAM-A1 PE = 2 SV = 1
Cluster-109720.2752-0F	4.3389	1.30E−06	NAC domain-containing protein 21/22 OS = Arabidopsis thaliana GN = NAC021 PE = 1 SV = 2
Cluster-109720.4140-1R	1.5252	0.013111	NAC domain-containing protein 100 OS = Arabidopsis thaliana GN = NAC100 PE = 2 SV = 1
Cluster-109720.6361-2F	4.4296	4.36E−15	NAC domain-containing protein 100 OS = Arabidopsis thaliana GN = NAC100 PE = 2 SV = 1
Cluster-109720.8457-2R	1.3415	0.014025	Protein FEZ OS = Arabidopsis thaliana GN = FEZ PE = 2 SV = 1

Cluster-109720.9952-2R	1.7674	0.00887	NAC domain-containing protein 90 OS = Arabidopsis thaliana GN = NAC090 PE = 2 SV = 1
Cluster-27434.1-0R	2.3281	8.32E−06	NAC domain-containing protein 77 OS = Oryza sativa subsp. japonica GN = NAC77 PE = 2 SV = 2
Cluster-27434.3-1R	3.9016	2.69E−06	NAC domain-containing protein 77 OS = Oryza sativa subsp. japonica GN = NAC77 PE = 2 SV = 2
Cluster-42202.0-2F	6.168	0.005724	NAC domain-containing protein 26 OS = Arabidopsis thaliana GN = NAC026 PE = 1 SV = 1
Cluster-84252.0-1F	5.1014	2.12E−08	NAC domain-containing protein 21/22 OS = Arabidopsis thaliana GN = NAC021 PE = 1 SV = 2
Cluster-87248.0-0F	5.9037	1.75E−06	NAC domain-containing protein 21/22 OS = Arabidopsis thaliana GN = NAC021 PE = 1 SV = 2
Cluster-95378.0-1F	5.2049	0.000583	NAC transcription factor 29 OS = Arabidopsis thaliana GN = NAC029 PE = 2 SV = 1
Cluster-109720.12111-0F	2.6329	2.90E−09	NAC domain-containing protein 83 OS = Arabidopsis thaliana GN = NAC083 PE = 1 SV = 1
Cluster-109720.671-1R	2.9521	0.040287	NAC domain-containing protein 73 OS = Arabidopsis thaliana GN = NAC073 PE = 2 SV = 1
Cluster-69913.0-0F	3.0322	0.017435	NAC transcription factor ONAC010 OS = Oryza sativa subsp. japonica GN = ONAC010 PE = 2 SV = 1
Cluster-100601.0-0F	1.5662	0.023745	NAC domain-containing protein 92 OS = Arabidopsis thaliana GN = NAC092 PE = 1 SV = 1
Cluster-109720.15034-0F	2.1432	0.001208	MACPF domain-containing protein At1g14780 OS = Arabidopsis thaliana GN = At1g14780 PE = 2 SV = 1
Cluster-109720.1896-1R	3.1755	6.31E−06	NAC transcription factor NAM-B2 OS = Triticum turgidum subsp. durum GN = NAM-B2 PE = 2 SV = 1
Cluster-109720.1897-2R	3.182	0.001046	NAC transcription factor ONAC010 OS = Oryza sativa subsp. japonica GN = ONAC010 PE = 2 SV = 1
Cluster-109720.1899-0R	3.7373	1.94E−08	NAC transcription factor ONAC010 OS = Oryza sativa subsp. japonica GN = ONAC010 PE = 2 SV = 1
Cluster-94493.0-0R	5.1998	6.18E−16	NAC domain-containing protein 73 OS = Arabidopsis thaliana GN = NAC073 PE = 2 SV = 1
Cluster-96318.0-1F	3.4374	4.72E−07	Transcription factor JUNGBRUNNEN 1 OS = Arabidopsis thaliana GN = JUB1 PE = 1 SV = 1
Cluster-99740.0-0R	3.1359	2.14E−09	NAC domain-containing protein 100 OS = Arabidopsis thaliana GN = NAC100 PE = 2 SV = 1
Cluster-109720.18046-1R	4.7169	4.79E−26	NAC transcription factor 56 OS = Arabidopsis thaliana GN = NAC056 PE = 2 SV = 1
Cluster-109720.2483-0R	9.651	8.63E−65	NAC transcription factor NAM-B2 OS = Triticum turgidum subsp. durum GN = NAM-B2 PE = 2 SV = 1
Cluster-109720.6052-2R	1.673	0.0396	NAC domain-containing protein 100 OS = Arabidopsis thaliana GN = NAC100 PE = 2 SV = 1
Cluster-109720.8393-2R	2.7368	1.68E−07	NAC domain-containing protein 48 OS = Oryza sativa subsp. japonica GN = NAC48 PE = 2 SV = 1
Cluster-109720.8394-2R	6.5986	1.29E−11	NAC domain-containing protein 48 OS = Oryza sativa subsp. japonica GN = NAC48 PE = 2 SV = 1
Cluster-109720.8395-1F	2.4845	0.000137	NAC domain-containing protein 48 OS = Oryza sativa subsp. japonica GN = NAC48 PE = 2 SV = 1
Cluster-109720.8396-2F	4.5677	1.02E−19	NAC domain-containing protein 48 OS = Oryza sativa subsp. japonica GN = NAC48 PE = 2 SV = 1

Cluster-51447.0-2F	4.0189	0.027623	NAC transcription factor 56 OS = Arabidopsis thaliana GN = NAC056 PE = 2 SV = 1
Cluster-96191.0-0R	2.5001	0.002667	Protein CUP-SHAPED COTYLEDON 1 OS = Arabidopsis thaliana GN = NAC054 PE = 1 SV = 1
Cluster-109720.15606-0R	1.5739	1.18E−05	NAC domain-containing protein 14 OS = Arabidopsis thaliana GN = NAC014 PE = 2 SV = 1
Cluster-109720.1900-0R	3.5299	1.72E−05	NAC transcription factor ONAC010 OS = Oryza sativa subsp. japonica GN = ONAC010 PE = 2 SV = 1
Cluster-109720.21428-0F	2.7119	3.20E−05	Putative NAC domain-containing protein 94 OS = Arabidopsis thaliana GN = ANAC094 PE = 3 SV = 1
Cluster-109720.6053-2F	1.8097	0.002364	NAC domain-containing protein 100 OS = Arabidopsis thaliana GN = NAC100 PE = 2 SV = 1
Cluster-109720.8482-1R	1.9612	6.55E−05	NAC domain-containing protein 68 OS = Oryza sativa subsp. japonica GN = NAC68 PE = 2 SV = 1
Cluster-109720.8483-0F	1.796	0.022013	NAC domain-containing protein 68 OS = Oryza sativa subsp. japonica GN = NAC68 PE = 2 SV = 1
Cluster-99233.0-2F	5.054	1.93E−12	NAC domain-containing protein 100 OS = Arabidopsis thaliana GN = NAC100 PE = 2 SV = 1
Cluster-99740.1-0R	3.1774	2.57E−08	NAC domain-containing protein 100 OS = Arabidopsis thaliana GN = NAC100 PE = 2 SV = 1
Cluster-109720.15903-0R	−4.602	1.51E−07	Putative NAC domain-containing protein 94 OS = Arabidopsis thaliana GN = ANAC094 PE = 3 SV = 1
Cluster-103492.0-0R	−7.1962	8.51E−06	NAC domain-containing protein 43 OS = Arabidopsis thaliana GN = NAC043 PE = 2 SV = 2
Cluster-83797.0-2R	−2.456	0.000344	Protein FEZ OS = Arabidopsis thaliana GN = FEZ PE = 2 SV = 1
Cluster-109720.10938-0R	−1.1285	0.012968	NAC domain-containing protein 8 OS = Arabidopsis thaliana GN = NAC008 PE = 2 SV = 1
Cluster-109720.14859-0R	−2.4357	1.47E−08	NAC transcription factor NAM-A1 OS = Triticum turgidum subsp. durum GN = NAM-A1 PE = 2 SV = 1

Table S4. The differentially expressed WRKY TFs between CK and DT.

GeneID	Log_2(DT/CK)	q-value	Gene Description
Cluster-101788.2-1R	1.699	0.008746	WRKY transcription factor 6 OS = Arabidopsis thaliana GN = WRKY6 PE = 1 SV = 1
Cluster-106814.0-2R	1.2263	0.020767	Probable WRKY transcription factor 28 OS = Arabidopsis thaliana GN = WRKY28 PE = 2 SV = 1
Cluster-109720.19148	3.0049	5.71E−08	Probable WRKY transcription factor 69 OS = Arabidopsis thaliana GN = WRKY69 PE = 2 SV = 1
Cluster-82758.0-2F	2.9458	0.027264	Probable WRKY transcription factor 70 OS = Arabidopsis thaliana GN = WRKY70 PE = 2 SV = 1
Cluster-91516.0-0R	4.8473	1.69E−08	WRKY transcription factor 22 OS = Arabidopsis thaliana GN = WRKY22 PE = 2 SV = 1
Cluster-91516.1-1F	2.2102	0.000378	Probable WRKY transcription factor 35 OS = Arabidopsis thaliana GN = WRKY35 PE = 2 SV = 1
Cluster-109720.11860-1F	1.1072	0.005435	Probable WRKY transcription factor 70 OS = Arabidopsis thaliana GN = WRKY70 PE = 2 SV = 1
Cluster-114727.0-1F	2.4197	0.0254	Probable WRKY transcription factor 31 OS = Arabidopsis thaliana GN = WRKY31 PE = 2 SV = 1
Cluster-109720.23905-0R	4.9281	4.43E−19	WRKY transcription factor 18 OS = Arabidopsis thaliana GN = WRKY18 PE = 1 SV = 2

Cluster-109720.9608-1R	3.5522	2.00E−15	Probable WRKY transcription factor 75 OS = Arabidopsis thaliana GN = WRKY75 PE = 2 SV = 1
Cluster-65261.20-0F	13.006	2.02E−19	Probable WRKY transcription factor 9 OS = Arabidopsis thaliana GN = WRKY9 PE = 2 SV = 1
Cluster-102616.0-2R	4.0844	1.19E−11	Probable WRKY transcription factor 2 OS = Arabidopsis thaliana GN = WRKY2 PE = 2 SV = 1
Cluster-109720.17078-2R	5.7468	2.49E−24	WRKY transcription factor 18 OS = Arabidopsis thaliana GN = WRKY18 PE = 1 SV = 2
Cluster-109720.3023-0F	4.899	0.000208	WRKY transcription factor 18 OS = Arabidopsis thaliana GN = WRKY18 PE = 1 SV = 2
Cluster-109720.3671-0R	1.3496	0.006896	WRKY transcription factor 6 OS = Arabidopsis thaliana GN = WRKY6 PE = 1 SV = 1
Cluster-27838.0-1R	12.492	1.11E−16	Probable WRKY transcription factor 47 OS = Arabidopsis thaliana GN = WRKY47 PE = 2 SV = 2
Cluster-85937.1-0R	1.5987	0.01037	Probable WRKY transcription factor 11 OS = Arabidopsis thaliana GN = WRKY11 PE = 2 SV = 2
Cluster-93467.0-1F	2.6851	6.26E−08	Probable WRKY transcription factor 2 OS = Arabidopsis thaliana GN = WRKY2 PE = 2 SV = 1
Cluster-97443.0-2F	1.0613	0.038167	Probable WRKY transcription factor 46 OS = Arabidopsis thaliana GN = WRKY46 PE = 2 SV = 1
Cluster-102086.0-1R	1.5088	0.01208	Probable WRKY transcription factor 11 OS = Arabidopsis thaliana GN = WRKY11 PE = 2 SV = 2
Cluster-109720.17113	1.003	0.026133	Probable WRKY transcription factor 65 OS = Arabidopsis thaliana GN = WRKY65 PE = 2 SV = 1
Cluster-109720.1902-2F	2.4913	4.68E−09	WRKY transcription factor 6 OS = Arabidopsis thaliana GN = WRKY6 PE = 1 SV = 1
Cluster-110435.0-0F	4.1727	2.59E−19	Probable WRKY transcription factor 24 OS = Arabidopsis thaliana GN = WRKY24 PE = 2 SV = 1
Cluster-116278.0-0F	2.81	5.24E−08	Probable WRKY transcription factor 3 OS = Arabidopsis thaliana GN = WRKY3 PE = 2 SV = 1
Cluster-94761.0-0R	1.8806	0.028143	WRKY transcription factor 22 OS = Arabidopsis thaliana GN = WRKY22 PE = 2 SV = 1
Cluster-109720.15502-0F	−1.3378	0.044339	Probable WRKY transcription factor 51 OS = Arabidopsis thaliana GN = WRKY51 PE = 1 SV = 1
Cluster-111267.0-0F	−2.6485	0.00011729	Probable WRKY transcription factor 57 OS = Arabidopsis thaliana GN = WRKY57 PE = 2 SV = 1
Cluster-111839.0-1R	−3.2876	4.57E−06	Probable WRKY transcription factor 35 OS = Arabidopsis thaliana GN = WRKY35 PE = 2 SV = 1
Cluster-109720.13624-0F	−1.3471	0.035057	Lipase-like PAD4 OS = Arabidopsis thaliana GN = PAD4 PE = 1 SV = 1
Cluster-109720.19349-0F	−3.5514	0.0011986	Probable WRKY transcription factor 54 OS = Arabidopsis thaliana GN = WRKY54 PE = 2 SV = 2
Cluster-109720.6123-0R	−3.3363	2.18E−06	Probable WRKY transcription factor 53 OS = Arabidopsis thaliana GN = WRKY53 PE = 1 SV = 1
Cluster-109720.6124-0R	−3.6436	8.32E−11	Probable WRKY transcription factor 70 OS = Arabidopsis thaliana GN = WRKY70 PE = 2 SV = 1
Cluster-78337.1-0F	−2.6445	2.25E−06	Probable WRKY transcription factor 9 OS = Arabidopsis thaliana GN = WRKY9 PE = 2 SV = 1
Cluster-109720.7417-1F	−3.4762	5.82E−06	Protein WRKY1 OS = Zea mays PE = 1 SV = 1
Cluster-111267.1-1F	−4.018	2.24E−07	Probable WRKY transcription factor 57 OS = Arabidopsis thaliana GN = WRKY57 PE = 2 SV = 1

Table S5. The differentially expressed HSF TFs between CK and DT.

GeneID	Log_2(DT/CK)	q-value	Gene Description
Cluster-58701.0-1R	6.7018	0.005199	Putative heat stress transcription factor A-6a OS = Oryza sativa subsp. japonica GN = HSFA6A PE = 3 SV = 1
Cluster-109720.11-1F	4.9929	1.06E−16	Heat stress transcription factor B-2b OS = Oryza sativa subsp. japonica GN = HSFB2B PE = 2 SV = 1
Cluster-109720.10-0R	3.4434	6.82E−07	Heat stress transcription factor B-2c OS = Oryza sativa subsp. japonica GN = HSFB2C PE = 2 SV = 1
Cluster-109720.20196-2R	2.89	0.002413	Heat stress transcription factor B-1 OS = Oryza sativa subsp. japonica GN = HSFB1 PE = 2 SV = 1
Cluster-109720.22375-0F	2.6862	0.000201	Heat stress transcription factor B-2a OS = Oryza sativa subsp. japonica GN = HSFB2A PE = 2 SV = 2
Cluster-109720.22376-1F	2.1551	0.019222	Heat stress transcription factor B-2a OS = Oryza sativa subsp. japonica GN = HSFB2A PE = 2 SV = 2

Table S6. All primers used in this study.

Name	Sequence
CdACTIN2-F	TCTGAAGGGTAAGTAGAGTAG
CdACTIN2-R	ACTCAGCACATTCCAGCAGAT
CdPIF3-F	GTACGACGATGACGATGAGC
CdPIF3-R	GCTGGTTGATCTACGGTCCA
CdPIF4-F	AGGACAGTTGCATACCGTGT
CdPIF4-R	ATGAGCTTCTACCAGCAGCA
CdWRKY22-F	AGAACGCCTTGCTCATCGTC
CdWRKY22-R	AGTGGACATGACGCTGCTTG
CdWRKY9-F	TCACTGACATGTGTGCCTCT
CdWRKY9-R	CTTCTTCTCCCGAGGCATCT
CdNAC092-F	GAGGAGCTCATCACGCACTA
CdNAC092-R	GGTCCTTGACGCAGAAGAAG
CdNAC21/22-F	TCTGCAGGGTGTTCTACAGG
CdNAC21/22-R	GTGGTCCAAAGCTAGGAGGT

plants

MDPI

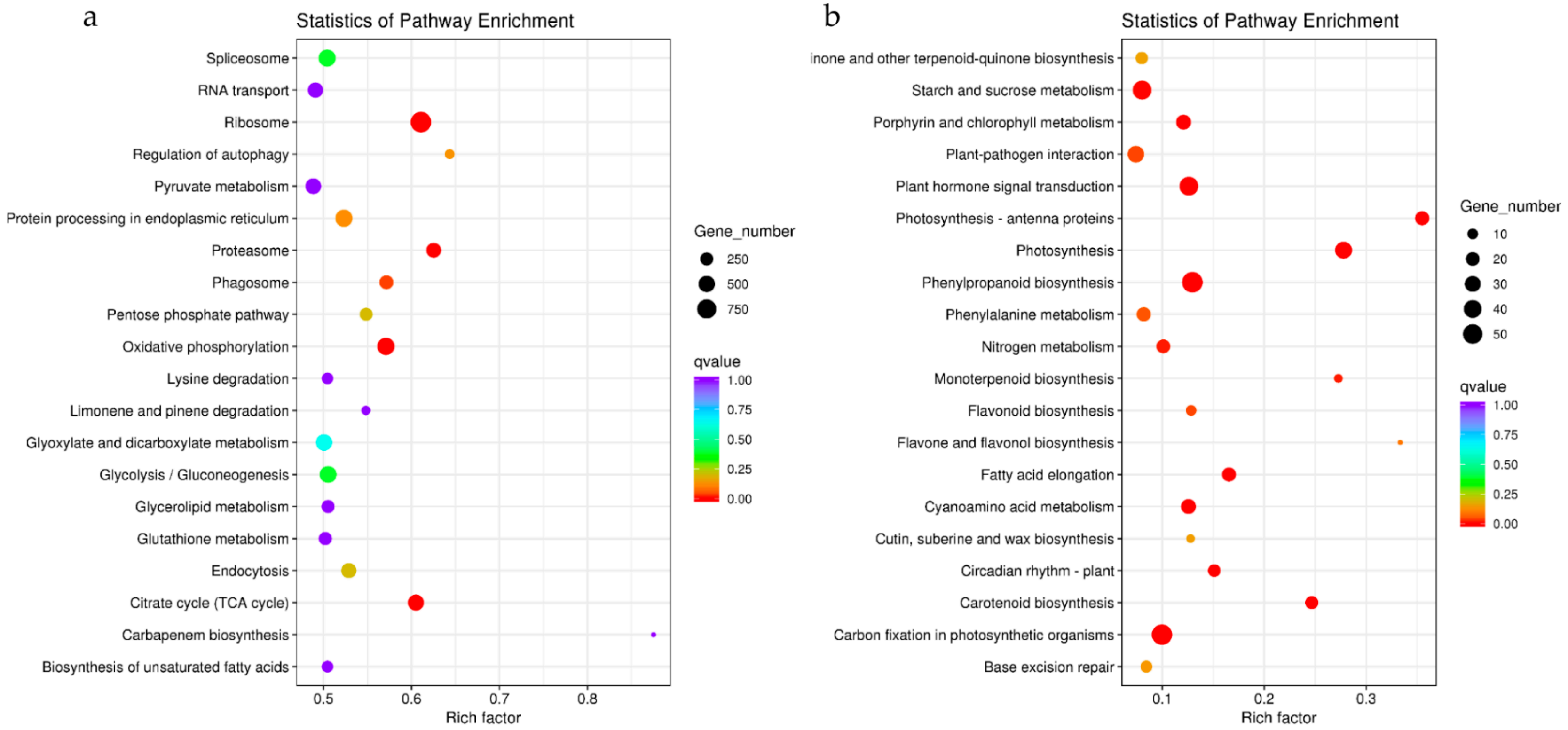

Figure S1. Kyoto encyclopedia of genes and genomes (KEGG) analysis of DEGs between CK and DT. (**a**) KEGG analysis of up-regulated DEGs; (**b**) KEGG analysis of down-regulated DEGs.

plants MDPI

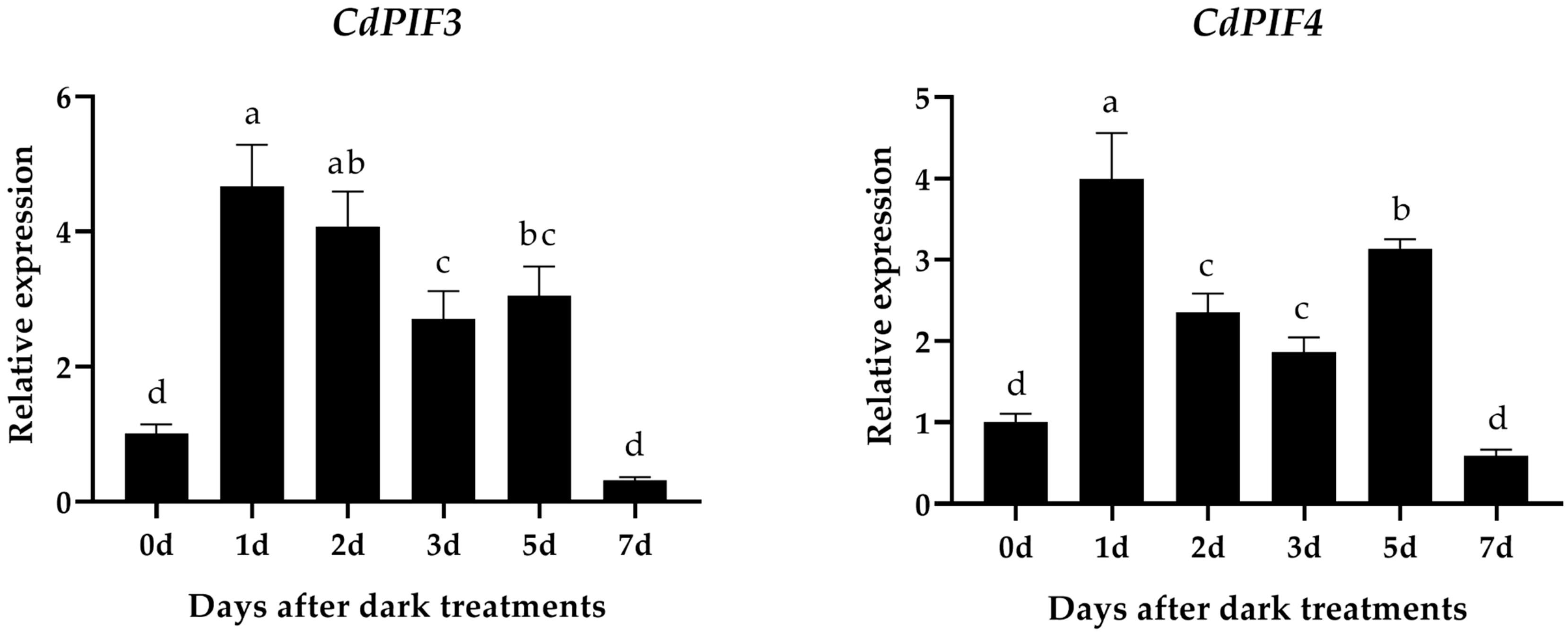

Figure S2. qRT-PCR analysis of *CdPIF3* and *CdPIF4* during dark-induced leaf senescence. Relative expression of (**a**) *CdPIF3*; (**b**) *CdPIF4* in the first and second leaves from bermudagrass after 1, 1, 2, 3, 5 and 7 d of dark treatments. Letters above error bars indicate significant difference ($p < 0.05$) using Tukey's HSD test. The error bars represent the SD value ($n = 3$).
